# Imaging Findings of Lupus Mastitis: A Systematic Review of Case Studies

**DOI:** 10.7759/cureus.79064

**Published:** 2025-02-15

**Authors:** Stephanie Nagy, Kitty Daniel, Marc M Kesselman

**Affiliations:** 1 Rheumatology, Nova Southeastern University Dr. Kiran C. Patel College of Osteopathic Medicine, Davie, USA; 2 Radiology, Mount Sinai Medical Center, Miami, USA

**Keywords:** breast lesion, cutaneous lupus erythematosus, discoid lupus erythematosus, lupus erythematosus profundus, lupus mastitis, lupus panniculitis, systemic lupus erythematosus

## Abstract

Lupus mastitis (LM) is a rare manifestation of systemic lupus erythematosus, resulting in an inflammatory process within the breast tissue. This condition presents diagnostic challenges due to its similarity to other breast pathologies, including infections and malignancies. Clinically, patients may exhibit symptoms such as breast pain, swelling, and erythema, often leading to further investigation through imaging and histopathological analysis. Given the absence of established clinical guidelines for LM, identifying the most effective imaging and laboratory techniques is essential for accurate diagnosis and treatment. In this paper, 32 patient case reports were analyzed, with 20 patients having previous diagnoses of lupus and 12 not previously diagnosed. The study population comprised 90.6% (n=29) women, with a mean age of 44 years and an average disease duration of 10 years. The majority of LM was unilateral, with 50% (n=16) of patients experiencing LM in the right breast, 31.2% (n=10) in the left breast, and 18.8% (n=6) bilaterally. The location varied to almost all quadrants of the breast. The rarity of LM contributes to clinician unawareness, often resulting in misdiagnosis and confusion. As there are currently no guidelines, in this study we examined the imaging findings of LM through a variety of modalities and reported a suggested clinical guideline to follow for the diagnoses of LM.

## Introduction and background

Lupus mastitis (LM) is a rare manifestation of systemic lupus erythematosus (SLE), resulting in an inflammatory process within the breast tissue. In 1883, Kaposi first described lupus panniculitis, a condition characterized by inflammation in the deep subcutaneous layer of the skin, leading to nodules or plaques [1,2]. LM is an uncommon presentation of lupus panniculitis in breast tissue. LM is commonly seen in patients with diagnosed SLE or discoid lupus erythematosus (DLE); however, rarely, LM may be the initial presentation of SLE or DLE before official diagnosis [3,4]. LM can present as single or multiple subcutaneous masses, unilaterally or bilaterally, which may be painful or palpable. The overlying skin can be unaffected or exhibit atrophy, lipoatrophy, hypertrichosis, hyperkeratosis, ulceration, erythema, thickening, scaling, and lesions [5,6]. Histologically, it most commonly presents with lymphocytic infiltrates and fat necrosis.

Given the higher prevalence of autoimmune conditions in women, LM is more commonly observed in middle-aged premenopausal women, typically around 40 years old. However, it can affect individuals between 18 and 70 years old, with significantly fewer cases reported in men [5,7]. In the literature, only five reported cases of LM occurred in men from 1983 to 2016 [8-12].

Due to the rarity of LM, incidence is often based on the finding of lupus panniculitis, which refers to inflammation of the subcutaneous fat and occurs in 2-3% of patients with SLE. LM is a subset of lupus panniculitis only affecting the breast tissue. There is currently no definitive incidence rate for LM [13,14].

The pathophysiology of LM is yet to be concretely discovered. Hypotheses believe that the inflammatory process of both the adaptive and innate immune systems impacts the skin overlying the breasts, resulting in visual changes from erythema, scaling, atrophy, ulcerations, and lesions. This theory is further supported by the presence of immune complexes, immunoglobulins of IgA, IgG, IgM, and complement factors of C3 and C4 within the basement membrane of the dermal-epidermal junction and blood vessels. This is the most supported hypothesis, as patients have been found to improve with the use of anti-inflammatory medications, including corticosteroids [3-6,15]. Some patients exhibit no visible breast changes, which is attributed to vasculitis [16]. Furthermore, trauma to the affected site through a biopsy is thought to worsen the condition and is advised to be avoided if diagnoses could be made through non-invasive imaging [5,17]. There is no current evidence linking LM with any other subsequent disease manifestation of lupus, such as lupus nephritis, as the pathophysiology significantly differs. 

While certain medications can induce SLE, there is no direct evidence linking these medications to the development of LM. LM is more likely to occur in the context of pre-existing SLE or DLE rather than being directly induced by certain medications. The literature primarily focuses on the systemic effects of drug-induced lupus, which typically includes musculoskeletal symptoms, serositis, and skin manifestations, but does not specifically address LM. Therefore, while these drugs can induce SLE, their role in causing LM remains unsubstantiated [18,19].

Due to the heterogeneous presentation of LM, clinicians consider differential diagnoses for patients at the time of presentation, including medullary breast carcinoma, inflammatory breast carcinoma, diabetic mastopathy, subcutaneous panniculitis-like T-cell lymphoma, and granulomatous mastitis. Therefore, histopathology is crucial to proper diagnoses [7,15].

Despite its clinical significance, LM remains poorly understood, and no standardized diagnostic guidelines currently exist. This study aims to bridge this gap by analyzing reported cases of LM to identify common clinical and imaging findings and propose a diagnostic framework for clinicians. We also recommend modifying existing clinical guidelines to acknowledge that an LM diagnosis fulfills the criteria for an SLE diagnosis, regardless of whether the additional requirements outlined in current guidelines are met.

## Review

Methods

Search Strategy

A systematic review was conducted using OVID, EMBASE, and Web of Science using the search term of only “Lupus mastitis.” To ensure the relevancy of the articles, those published between 2000 and 2024 were assessed to ensure that a larger number of studies could be reviewed due to the rarity of this condition. The articles were analyzed in a step-wise process, first evaluating the title, abstract, and study design. Full texts were then used to analyze the availability of articles and ensure patients who only had LM without a secondary autoimmune condition were analyzed. Nova Southeastern University’s library database was used to access databases and full-text articles.

Selection Criteria

**Figure 1:**
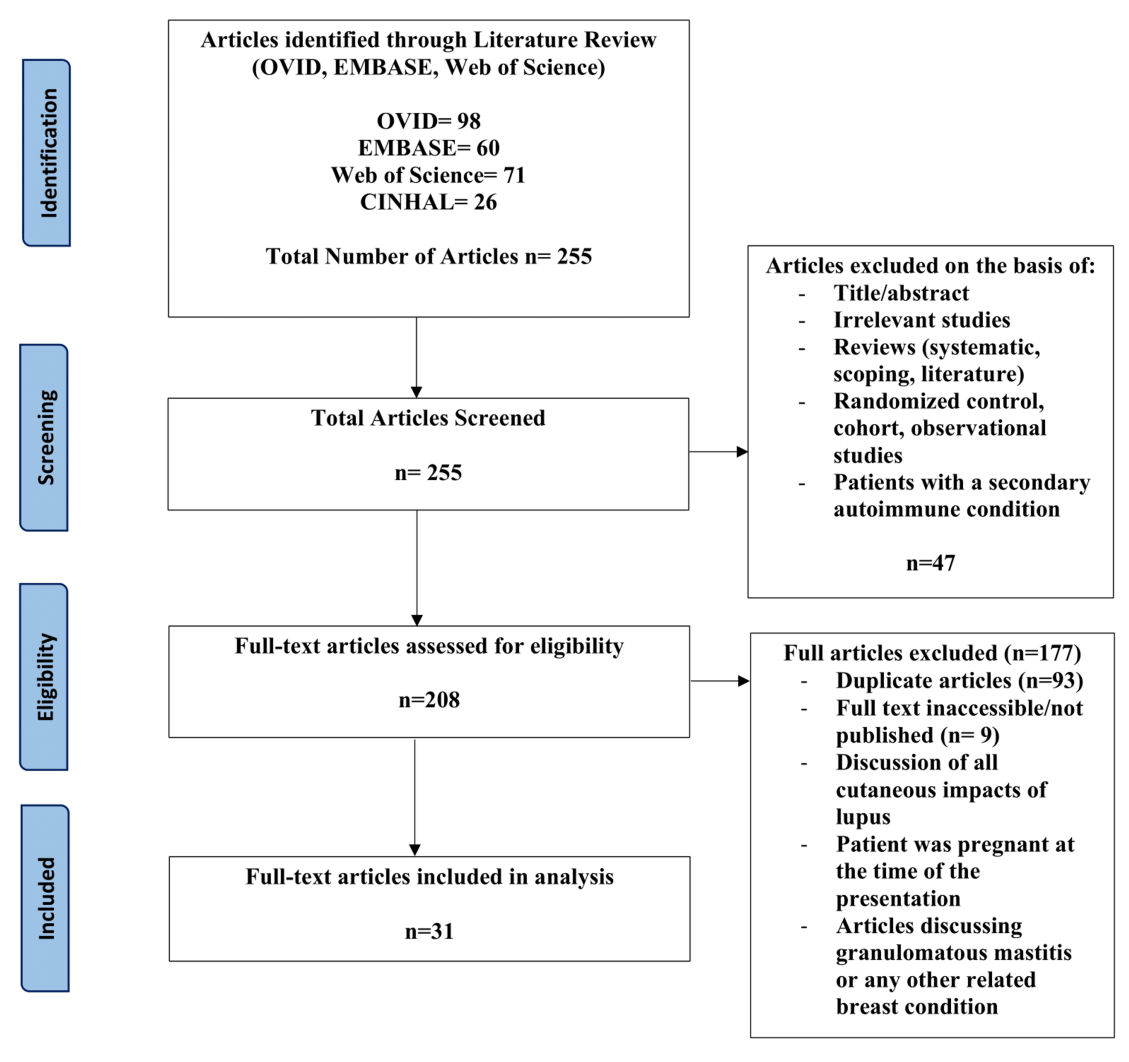
PRISMA flow diagram for selection criteria PRISMA: Preferred Reporting Items for Systematic Reviews and Meta-Analyses

The study designs included only case studies. Exclusion criteria included study designs of literature, systematic or scoping reviews, randomized control trials, cross-sectional studies, observational studies, cohort prospective/retrospective studies, and animal studies. Abstracts without full text were excluded to limit the analysis to fully described patient characteristics and associated imaging. Articles were removed if the patient had a secondary autoimmune condition or pregnancy to limit confounding variables and articles that discussed other forms of mastitis. Preferred Reporting Items for Systematic Reviews and Meta-Analyses (PRISMA) were used to develop a flow diagram of the selection criteria for reproducibility (Figure 1) [20].

Results

**Figure 2:**
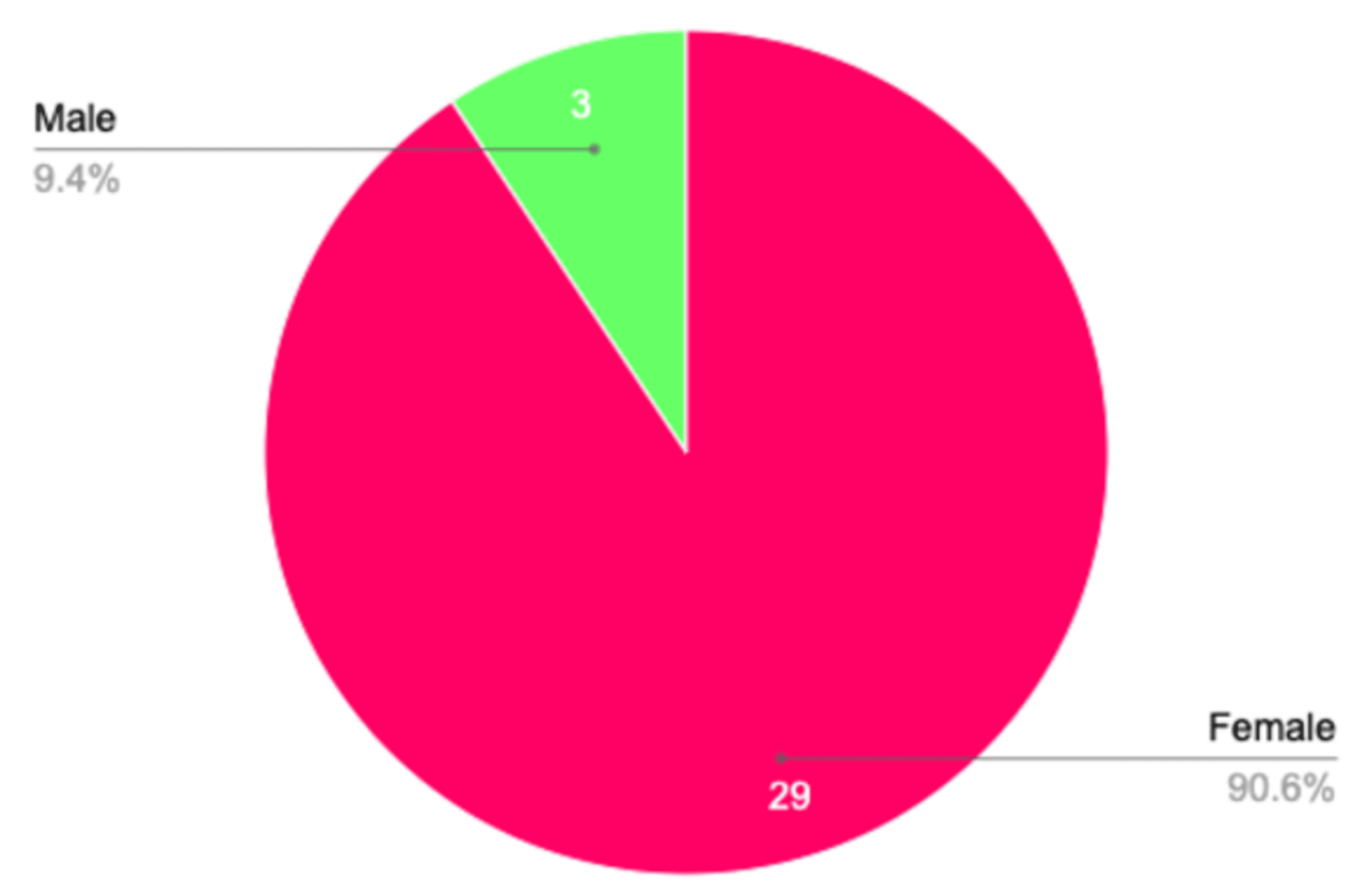
Comparison of female to male patients

In total, 255 articles were populated between the databases of OVID, EMBASE, Web of Science and CINHAL, with 30 articles being selected after first and second-tier screening. Table 1 indicates the characteristics of the 32 patients with LM that were analyzed among the articles; 29 (90.6%) were female, with only three (9.4%) being seen in male (Figure 2). The age ranged from 24 to 66 years old, with a mean age of 44. Twelve patients were not previously diagnosed with any form of lupus at the time of presenting with LM. Following a diagnosis of LM, four of the 12 patients were diagnosed with lupus (one SLE, three DLE). Out of the remaining studies with previous diagnosis, 15 reported disease duration with a range of 3 to 20 years, and the mean duration was 10 years. Ethnicity was reported in only nine of the studies (one Asian, three White, and five African-American).

**Table 1:** Characteristics of patients and study findings ESR: Erythrocyte sedimentation rate; ANA: antinuclear antibody; ANCA: antineutrophil cytoplasmic antibodies; DLE: discoid lupus erythematosus; SLE: systemic lupus erythematosus; CLE: cutaneous lupus erythematosus; dsDNA: double-stranded DNA; WBC: white blood cell

Title	Patient Age and Gender (M/F)	Ethnicity	Disease Duration	History of Lupus	Lab Results	Location	Physical Appearance	US Findings	Mammogram Findings	MRI Findings	CT Findings	Biopsy/Histological Findings	Treatment Received
Guo et al. [21]	27 F	Asian (Chinese)	No previous diagnosis	No previous diagnosis.	ESR=57mm; IgG =17.33 g/L; IgA = 3.95 g/L; IgM normal; C3 and C4 normal; ANA normal; ANCA negative ; ribosomal P protein positive	Upper inner quadrant on the right breast	Skin thickening, erythematous; nine months later: the patient declined initial treatment and returned with a palpable mass, ulceration, lymphadenopathy	Subcutaneous, hyperechoic 3cm lesion; abundant peripheral and internal blood flow signals; no distinct mass or calcification. Nine months later: subcutaneous, hyperechoic lesion at 6.5cm; increased blood flow to the hyperechoic area	Not completed.	Nine months later: mass with dimensions of 7.7×6.9×3.2 cm; T1 and T2 intensity type 1 kinetic curve in the right breast; Linear region of intensity extending from the right upper inner breast to left upper inner breast	Not completed	Fat lobule necrosis; mixed lymphoplasmacytic and histiocytic aggregates; granulomas; vasculitis	Corticosteroids; hydroxychloroquine leading to complete remission
Taslicay et al. [22]	58 F	Not reported	11 years	DLE	ANA elevated	Subareolar area of the left breast	Palpable mass and swelling of the left breast. Two years later: the patient was not adherent to medications presented again with painful palpable masses in the left breast and left arm	Diffuse edema; irregular hypoechoic areas; acoustic shadowing	8 cm mass in the subareolar area of the left breast; skin thickening; diffuse axillary lymphadenopathy. Two years later: skin thickening; newly developed focal asymmetry; diffuse microcalcification; slight regression in the previous focal asymmetry	Fat necrosis; irregular heterogeneous, and rim-enhancing fat-containing masses. Two years later: T1 and T2 hyperintense fat-containing multiple masses with heterogeneous and rim enhancement indicating fat necrosis	Focal masses with increased densities, areas of fat necrosis. Two years later: fat necrosis	Lymphocytic. perineuronal, perivascular, and periductal lymphoid infiltrates; mononuclear cell infiltration with germinal center formation; hyaline-type fat necrosis; lymphocytic vasculitis	Patient used hydroxychloroquine, metronidazole, doxycycline tablets, permethrin, and sodium-sulfacetamide during flare ups. Two years later: Restarted on hydroxychloroquine = complete remission with increased microcalcifications
Wang et al. [23]	26 F	Not reported	9 years	SLE	Not reported	Bilateral mass	No physical findings only palpable masses	Irregular hypoechoic areas bilaterally (right breast = 85×13 mm left breast= 52×13 mm); poorly defined borders; coarse, strong echoes; blood flow in the hypoechoic area	Not completed.	Double breast-occupying lesions	Not completed.	Interstitial fibrosis; Vitreous degeneration; fat necrosis; microcalcifications; lymphocyte infiltration around the lobules and stroma of both breasts	Hydroxychloroquine, corticosteroids
Pimentel et al. [24]	35 F	Not reported	Not reported	SLE	Not reported	Right outer lower breast	Swollen hardened breast	Lobulated vascularized 5 cm mass with liquid areas	Not completed	Not completed.	Not completed.	Perilobular and periductal lymphoid infiltrate; lymphocytic vasculitis	Hydroxychloroquine, prednisolone remission in one month
Oktay et al. [25]	37 F	Not reported	No previous diagnosis	Diagnosed with SLE following presentation	Hemoglobin = 10.9 g/dL; white blood cells = 2,690/mm^3^; neutrophils = 53.5%; platelets = 127,000/mm^3^, anti-ribosomal antibodies positive, ANA positive	Bilateral breasts	Palpable breast masses	Axillary lymphadenopathy Ill-defined isoechoic masses, with acoustic shadows; Dystrophic calcifications	Diffuse calcifications indicating fat necrosis	Not completed	Not completed	Not completed	Hydroxychloroquine, corticosteroid
Jimenez-Anton et al. [26]	50 F	Not reported	No previous diagnosis	No previous diagnosis	ANA titer= 1/160	Right breast	Erythema, scales, and periareolar excoriation	Not completed	Not completed	Not completed	Not completed	Epidermal atrophy; perivascular lymphocytic infiltrate; vacuolar degeneration of the basal layer; accumulation of mucin; lupus band with linear IgG deposits; IgM deposits in the dermal-epidermal junction	Hydroxychloroquine, corticosteroid remission after one year
Mazeda et al. [27]	34 F	Not reported	Not reported	SLE	Not reported	Right lower outer quadrant. One year later: Bilateral lesion	Hard, tenderness, warmth	Irregularly defined lobulated mass; Vascularized mass 5 × 5 cm in size	Not completed	Not completed	Not completed	Fibrotic areas; abscesses; ductitis lesions; lymphatic lobulitis; vasculitis	Initial treatment: NSAID, antibiotics. Two weeks later: hydroxychloroquine, prednisolone. One year later: rituximab then reaching remission
Sharma et al. [28]	42 F	Not reported	No previous diagnosis	No previous diagnosis	Not reported	Bilaterally	Pain, tenderness	14 × 11 × 8-mm oval mass; hyperechoic	Not completed	Not completed	Not completed	Fat necrosis; Mixed nodular lymphoplasmacytic and histiocytic aggregates; Karyorrhectic debris; fibrinoid necrosis; microcalcifications; histiocytes positive for CD68 staining	Corticosteroids remission in three months
Tanaka et al. [29]	46 F	Not reported	20 years	SLE	Not reported	Right breast middle-inner	Firmness, crusting of skin	Skin thickening; Diffuse hypoechoic area with calcifications	Coarse calcification	Skin thickening; Skin atrophy; Diffuse enhancement effects of the right mammary gland	Not completed	Lymphocytic infiltration	None, follow-up observation
Vilas-Sueiro et al. [15]	60 F	Not reported	3 years	DLE	ANA titers 1:80 positive DsDNA 1:10 positive	Lower central left breast	Deep and firm erythematous subcutaneous nodule, no skin changes,	Increased density and irregular breast tissue, skin thickening	Not completed	Not completed	Not completed	Lymphocytic infiltration; Hyalinized fat necrosis; IgA, IgG, IgM, and C3 granular deposition	Hydroxychloroquine
Voizard et al. [6]	Case 1: 64 F Case 2: 66 F	Case 1: White Case 2: White	Case1: No previous diagnosis Case 2: No previous diagnosis	Case 1: No previous diagnosis Case 2: No previous diagnosis	Case 1: Six months later: elevated ANA	Case 1: Upper central right breast. Case 2: Inferior central right breast	Case 1: Skin thickening, erythematous/blue discoloration. Six months later: Extensive cutaneous lesions. Case 2: Erythema, mass in inframammary fold	Case 1: Diffuse hyperechogenic, skin thickening. Six months later: Axillary lymphadenopathy. Case 2: Skin thickening, non-specific hyperechoic infiltration of the subcutaneous fat and parenchyma	Case 1: Trabecular thickening. Six months later: Skin and trabecular thickening. Case 2: mild skin thickening in the right inframammary fold	Case 2: Skin thickening with enhancement of the dermis and subdermal regions	Not completed	Case 1: Hyaline fat necrosis; Lymphocytic infiltration in lobules and stroma; CD3-positive T-lymphocytes; CD20-positive B-lymphocytes. Six months later: perivascular and periductal non-specific lymphocytic infiltrate Follicular hyperplasia in lymph nodes. Case 2: Lymphoplasmacytic perineural infiltration; lymphocytic vasculitis in the deep dermis; hyalinization and sclerosis of adipose tissue and deep dermal mucin deposition	Case 1: Hydroxychloroquine. Case 2: Spontaneous remission
Thapa et al. [11]	39 M	Not reported	No previous diagnosis	Diagnosed with DLE following presentation	SSA/Ro positive ANA titer (1:40) positive	Bilaterally. Left supra-areolar region. Right upper outer quadrant	Tender, subcutaneous masses	Hyperechoic; Internal vascularity; 25 × 7 mm in the left breast and 22 × 9 mm in the right breast	Bilateral dense masses ; 3 × 3 cm mass in the left breast; 2 × 2 cm mass in the right breast	Fat stranding with overlying skin thickening hyperintense on T2 weighted; 29 × 24 mm mass in left breast; 13 × 8 mm mass in right breast; Heterogeneous enhancement; Type I kinetic enhancement curves	Not completed	Lymphoplasmacytic cell infiltration; Sclerosis; Intimal edema; Lymphocytic infiltration	Hydroxychloroquine, prednisolone
Kim et al. [30]	65 F	Not reported	Not reported	CLE	Not reported	Right breast upper outer area	Skin thickening	Focal asymmetry in the inner breast and upper outer quadrant, skin thickening	Not completed	Not completed	Not completed	Not completed	Not reported
Kinonen et al. [5]	Case 1: 58 F Case 2: 52 F	Case 1: African American Case 2: African American	Case 1: 10 years Case 2: Not reported	Case 1: SLE Case 2: DLE	Not reported	Case 1: Right upper breast. 1 year later: Upper center left breast. Case 2: Left upper and central breast	Case 1: Firm, palpable mass	Case 1: Ill-defined, hyperechoic, 2.6-cm lesion, vascularity. One year later: Similarly to initial presentation	Case 1: Ill-defined, soft tissue density. 1 year later: Similarly to initial presentation. Case 2: Focal irregular density, microcalcifications	Not completed	Not completed	Case 1: Hyaline fat necrosis; Lymphocytic infiltration in subcutaneous fat, with germinal center formation; CD20^+^ B-cell follicles; CD3^+^, CD5^+^, CD4^+^, CD8^+^ T cells. 1 year later: Lymphoplasmacytic infiltrate of lobules and septa with vascularity. Case 2: lymphocytic infiltration of the adipose tissue in perivascular and periductal regions	Case 1: Hydroxychloroquine Case 2: Not reported
Lucivero et al. [31]	47 F	Not reported	10 years	SLE	SSA/Ro Antibody positive	Upper and lower quadrants of right breast	Firm, painless mass	Irregularly defined vascularized; Hypoechoic nodule 15 mm	Not completed	Not completed	Not completed	Dysplastic ductal cells; Macrophages with a foamy cytoplasm; Necrosis of adipose tissue; Chronic granulomatous inflammation; Mammary gland dysplasia with simple cysts that are coated with flattened epithelial ductal cells; Dense aggregates of lymphocytes and monocytes with plurinucleate giant cells; Stromal fibrosis; Hyaline degeneration; Vascularity in basal structures; Increased thickness of blood vessels; CD20-positive B lymphocytes; IgG-secreting plasma cells; Few CD4 and CD8 T cells; CD68-positive monocytes and macrophages; C3 in vascular and epithelial basal membranes	Previous episodes of LM treated with antibiotics and anti-inflammatory medications, no specified. Current treatment for flare up, not mentioned.
Wang et al. [32]	28 F	Not reported	13 years	SLE	Not reported	Bilaterally	Bilateral mastalgia; lump sensation	Ill-defined; hypoechoic areas	Multifocal, coarse calcifications	Not completed	Not completed	Coarse dystrophic calcification; Fatty necrosis; perivascular lymphocyte infiltration	Corticosteroids remission within one week
Mosier et al. [33]	40 F	African-American	20 years	DLE	Not reported.	Left breast	Painful, palpable	Dystrophic calcifications	Coarse; central calcifications	Hypointense; coarse calcifications; irregular; thick rim of enhancement	Not completed	Hyalinization; Lymphocytic inflammation; necrosis	Hydroxychloroquine with topical steroids
Warne et al. [34]	34 F	Not reported	7 years	SLE	Not reported	Right breast upper outer	Swelling and pain. Two years later: Patient returned with swelling and pain and peau d’orange with no palpable mass	Irregular hypoechoic mass; Diffusely oedematous. Two years later: 7 cm mass in the upper outer quadrant; breast edema; axillary lymphadenopathy	Increased density	Not completed	Not completed	Hyaline fat necrosis; calcification; aclerosis; Lymphocytic infiltrates in the Periductal, perivascular and perilobular; Lymphocytic vasculitis; interstitial fibrosis. Two years later: Similar findings to initial biopsy	The patient developed Pseudomonas infection at the biopsy site as a result steroids were not recommended and the patient was started on antibiotic only
Wani et al. [35]	24 F	Not reported	13 years	SLE	Normochromic normocytic anemia; Leucopenia; Albumin = 2.6 gm/dl; Mild elevation of transaminases; ESR = 115 mm; serum ferritin level = less than 2000 μg/ dl.	Left breast three months later: Right breast	Palpable lumps in the left breast, largest reported to be 4x3cm with axillary lymphanopathy. Three months later: Pain and swelling in right breast; Lumps reported in both breasts	Diffuse calcifications; Acoustic shadowing	“Unusual” calcifications	Not completed	Calcifications in breasts	Degenerated fat cells; lymphocytes; foci of calcification	Observed
Fernendez-Flores et al. [8]	42 M	Not reported	No previous diagnosis	Did not meet criteria for diagnosis all antibodies negative	Not reported	Left breast	Diffuse moderate enlargement of the left breast with a red; scaly plaque around the nipple	Not completed	Not completed	Not completed	Not completed	Mixed lobular and septal lymphocytic panniculitis; lymphocytic infiltration with lymphoid follicles; vasculitis; vacuolar alteration with a smudge appearance along the dermoepidermal junction; thickened basement membrane; mixed population of B cells-CD20+; T cells -CD3+, CD4+, and CD8– and histocytes -CD68+; polyclonal rearrangement of IgH, CDR2, CDR3, and TCR-gamma	Not reported
Arsenovic et al. [36]	33 F	Not reported	3 years	SLE	WBC= 11.2×10^9^/L; ESR= 46-80mm/h; dsDNA antibody level elevated =98 IU/mL; Urea=9.5mmol/L; Creatinine=156 µmol/L	Right upper quadrant breast	Tenderness and firm lump with a fluctuant center, 7×5×3.5 cm	Heterogeneous mass with multifocal; coarse calcifications; Poorly defined margins	Not completed	Not completed	Not completed	Atrophic epidermis; fat necrosis; lymphocytic infiltrate; microcalcification; fibrinoid necrosis; mononuclear inflammatory infiltrate; lymphocytic vasculitis	Prednisone
Sanders et al. [37]	31 F	African American	No previous diagnosis	Did not meet criteria for diagnosis	Not reported.	Right upper outer quadrant	Painless palpable thickening and erythema of the skin	Skin thickening; subcutaneous hyperechogenicity without a focal mass	Skin thickening; Increased subjacent stromal density; lymphadenopathy	Extensive skin thickening in the right; moderate persistent and plateau enhancement (Type I⁄II kinetics); bilateral axillary and subpectoral lymphadenopathy	Not completed.	Atrophic changes; segmental perivascular lymphoplasmacytic infiltrate of the deep dermal arteries; hyaline fat necrosis	Not reported
Fernandez-Torres et al. [38]	57 F	Not reported	No previous diagnosis	No previous diagnosis	Not reported	Left breast	Erythematous plaque; ill-defined; orange-peel; rubbery; deep; stone-like areas; superficial telangiectasia; intense atrophy	Not completed	Conducted no abnormalities noted	Not completed	Not completed	Lymphocytic vasculitis; Lymphocytic infiltrate forming lymphoid follicles with germinal centers; fibrinoid degenerative; coarse calcifications in the reticular dermis	Oral antimalarials initiated but, shortly discontinued due to adverse reaction and began on Dapson
Crevitis et al. [9]	39 M	Not reported	No previous diagnosis	Diagnosed with DLE following workup	Anti-SSA positive ANA titer of 1:40	Bilateral; left supra-areolar region; right upper outer quadrant	Tender subcutaneous masses 3x3cm and 2x2cm	Ill-defined, hyperechoic; Subcutaneous mass with internal vascularity measuring 25 × 7 mm in left and 22 × 9 mm in the right	Ill-defined; dense mass	Focal areas of marked fat stranding with overlying skin thickening measuring 29 × 24 in the left and 13 × 8 mm in the right; areas marked as hyperintense on T2 weighted fat-suppressed sequence; Heterogeneous enhancement; type I kinetic enhancement curves	Not completed.	Fibrofatty infiltrated with lymphoplasmacytic cells arranged in a lobular and focally septal distribution; Sclerosis; intimal edema; lymphocytic infiltration	Prednisolone and hydrochloroquine
Georgian-Georgian-Smith et al. [16]	44 F	Not reported.	16 years	SLE	Not reported	Left breast	Painful, erythema	Not completed	Curvilinear and coarse calcifications; Fat necrosis; Diffuse increased density of fibroglandular tissues	Not completed	Not completed	Necrotizing vasculitis; Fat necrosis Microcalcifications; mixed population of T and B lymphocyte; Perivascular lymphocytic infiltrate; dense stromal fibrosis; microcalcifications	Prednisone but could not tolerate medication eventually lead to a mastectomy due to severe pain
Bachmeyer et al. [39]	30 F	Not reported	11 years	DLE	Not reported	Right breast	Painful, erythema	Not completed	Curvilinear and coarse calcifications	Not completed	Not completed	Deposits of IgG, IgM, and C3 along the dermoepidermal junction; Lymphocytic infiltrate; Voluminous calcifications	Prednisolone and hydroxychloroquine
Nigar et al. [40]	40 F	Not reported	12 years	SLE	Elevated ANA titers 1:5120; Elevated anticardiolipin antibodies; Anti-SS-A positive	Right breast	Tender, lump.	Eight years later: low echogenicity; minimal vascularity	1 year later: 20-mm irregular mass	Not completed.	Not completed.	One year later: dense lymphoid infiltrates Reactive lymphoid follicle; Fibrosis; Densely hyalinized breast stroma with ‘onion-skin’ type of concentric fibrosis around the local vessels and into breast ducts and lobules; Focal hyaline fat necrosis. 8 years later: Dense lymphoplasmacytic infiltrate	Not reported
Carducci et al. [41]	62 F	Not reported.	No previous diagnosis	Diagnosed with DLE following workup	ANA titer of 1:40	Right breast upper quadrant	Painful, erythema, nodules	Skin thickening; hyperechoic; poorly defined; Absence of flow signal	Increased density; hypodiaphania	Not completed	Not completed	Fat necrosis	Antimalarials
Chen et al. [13]	29 F	African- American	Not reported	SLE	Lupus anticoagulant positive	Left breast, upper inner quadrant	Round, nontender, 5 cm nodule	Ill-defined; isoechoic; heterogeneous mass	Not completed.	Not completed	Not completed	Lymphocytic infiltration; plasma cell infiltrates	Not reported
Sabate et al. [42]	33 F	White	7 years	SLE	Not reported.	Left breast, upper quadrant	Nontender, fixed, mass	Echogenic mass with ill-defined margins; Skin thickening	2-cm irregular mass; Ill-defined margins involving the subcutaneous fat pad of the left breast; Skin thickening; Subtle retraction on the adjacent superficial parenchymal gland	Heterogeneous mass involving the subcutaneous fat pad with a peripheral zone of low signal intensity and a central area of high signal intensity Irregular margins; Rim enhancement; Skin thickening; Subtle parenchymal distortion	Not completed.	Hyaline necrosis; fat necrosis; perivascular lymphocytic inflammation; vasculitis	Corticosteroids

**Figure 3:**
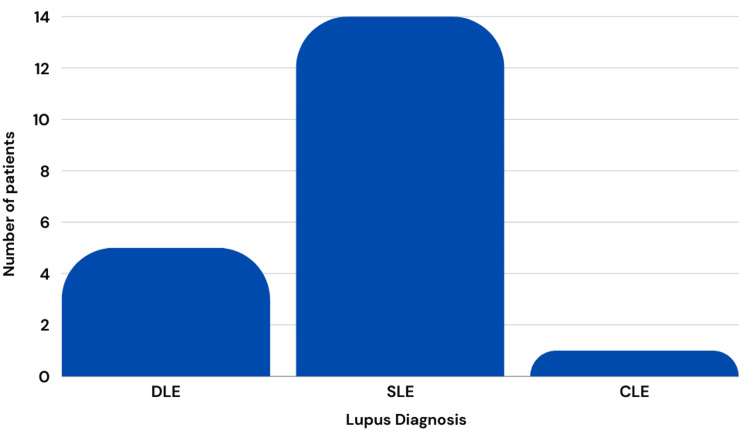
Type of lupus diagnosis among those with previous diagnosis SLE: Systemic lupus erythematosus; CLE: cutaneous lupus erythematosus; DLE: discoid lupus erythematosus

**Figure 4:**
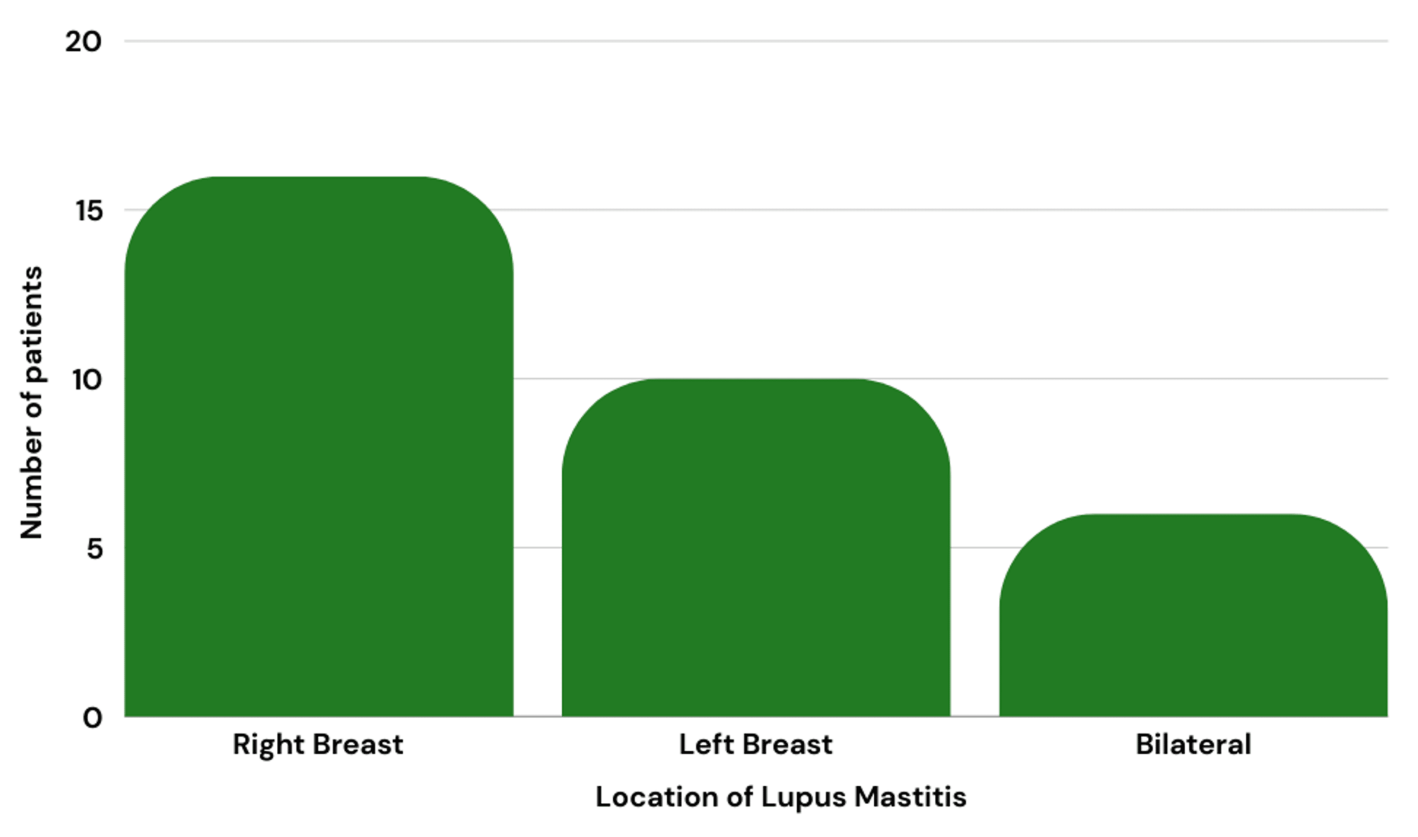
Location of lupus mastitis

Of the patients who were previously diagnosed, five were diagnosed with DLE, 14 with SLE, and one with CLE (Figure 3). Figure 4 represents the location of the initial breast lesions, with 16 (50%) on the right breast, 10 (31.2%) on the left breast, and 6 (18.8%) bilaterally.

The exact location within the breasts varied greatly, with masses in the right breast found in the upper inner, upper outer, upper central, lower central, lower outer, and middle inner quadrants versus those in the left breast seen in the subareolar, supraaerolar, lower central and upper central quadrants. Table 2 summarizes the common physical presentations of LM from the studies analyzed within ultrasound, mammogram, MRI, CT, and biopsy.

**Table 2:** Summary of physical and imaging findings of lupus mastitis

Physical findings	Ultrasound findings	Mammogram findings	MRI findings	CT findings	Biopsy findings
Skin thickening; erythematous; palpable mass; ulceration; lymphadenopathy; edema; scaling; crusting; warmth; excoriation; firmness; rubbery; stone-like; peau d’orange; cutaneous lesions; atrophy; telangiectasia [5, 6, 8, 9,11,15,16,20-42]	Hyperechoic lesion; hypoechoic lesion; isoechoic lesion; irregular; ill-defined; axillary lymphadenopathy; focal asymmetry; poorly defined borders; lobulated; vascularization; skin thickening; calcifications; edema; acoustic shadowing; strong echoes [5,6,9,11,15,21-25,27-29,30-38,40-42]	Skin thickening; trabecular thickening; axillary lymphadenopathy; calcifications; increased density [6,9,11,22, 25, 29, 33, 37, 42]	Type 1 kinetic curve; fat necrosis; T1 and T2 hyperintensity; rim-enhancing lesions; skin thickening; skin atrophy; diffuse enhancement; heterogeneous enhancement; fat stranding; hypointensity; calcification [6,9,11,21, 22, 23, 29, 33, 37, 42]	Increased density; fat necrosis [22, 35]	Fat necrosis; lymphoplasmacytic aggregates; lymphocytic infiltration; histiocyte aggregates; mononuclear cell infiltration; macrophages with a foamy cytoplasm; dysplastic ductal cells; mammary gland dysplasia with simple cysts; lymphoid follicles with germinal centers; vasculitis; granulomas; interstitial fibrosis; stromal fibrosis; calcification; epidermal atrophy; hyalinization; sclerosis; ductitis lesions; karyorrhectic debris; vacuolar alteration with a smudge appearance; accumulation of mucin; IgG, IgM, IgA deposits; C3 deposits; CD68 monocytes and macrophages; CD3, CD4, CD5, CD8 t cell deposit; CD 20 b cells deposit [5,6,8,9,11,15,21-24, 26-29, 31-38, 40-42]

**Figure 5:**
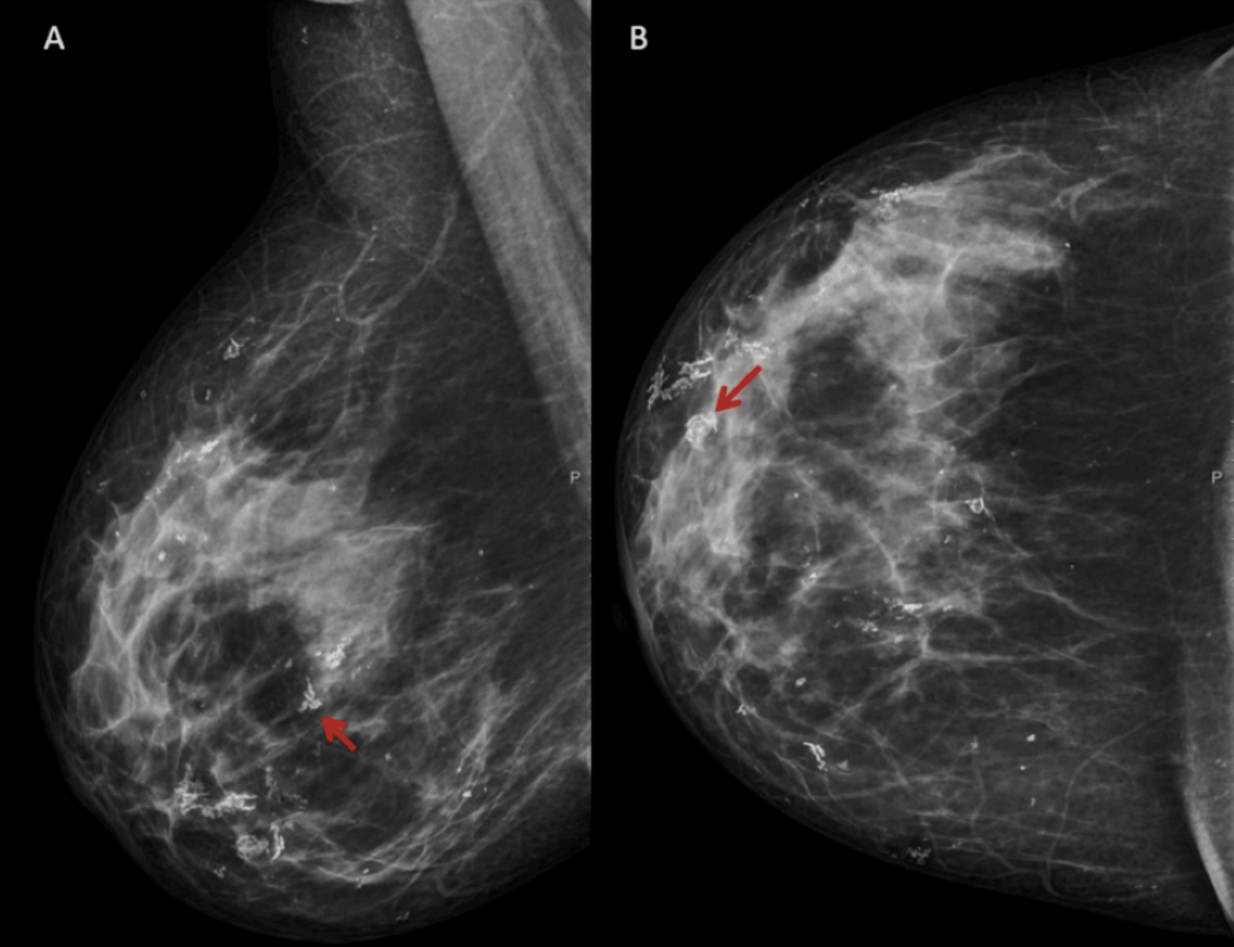
Oblique lateral (A) and craniocaudal (B) mammograms showing coarse and diffuse calcifications within lupus mastitis (red arrows) Image credit: Magalhaes et al. [43] (licensed under Creative Commons Attribution-NonCommercial-ShareAlike 4.0 International License; http://creativecommons.org/licenses/by-nc-sa/4.0/)

**Figure 6:**
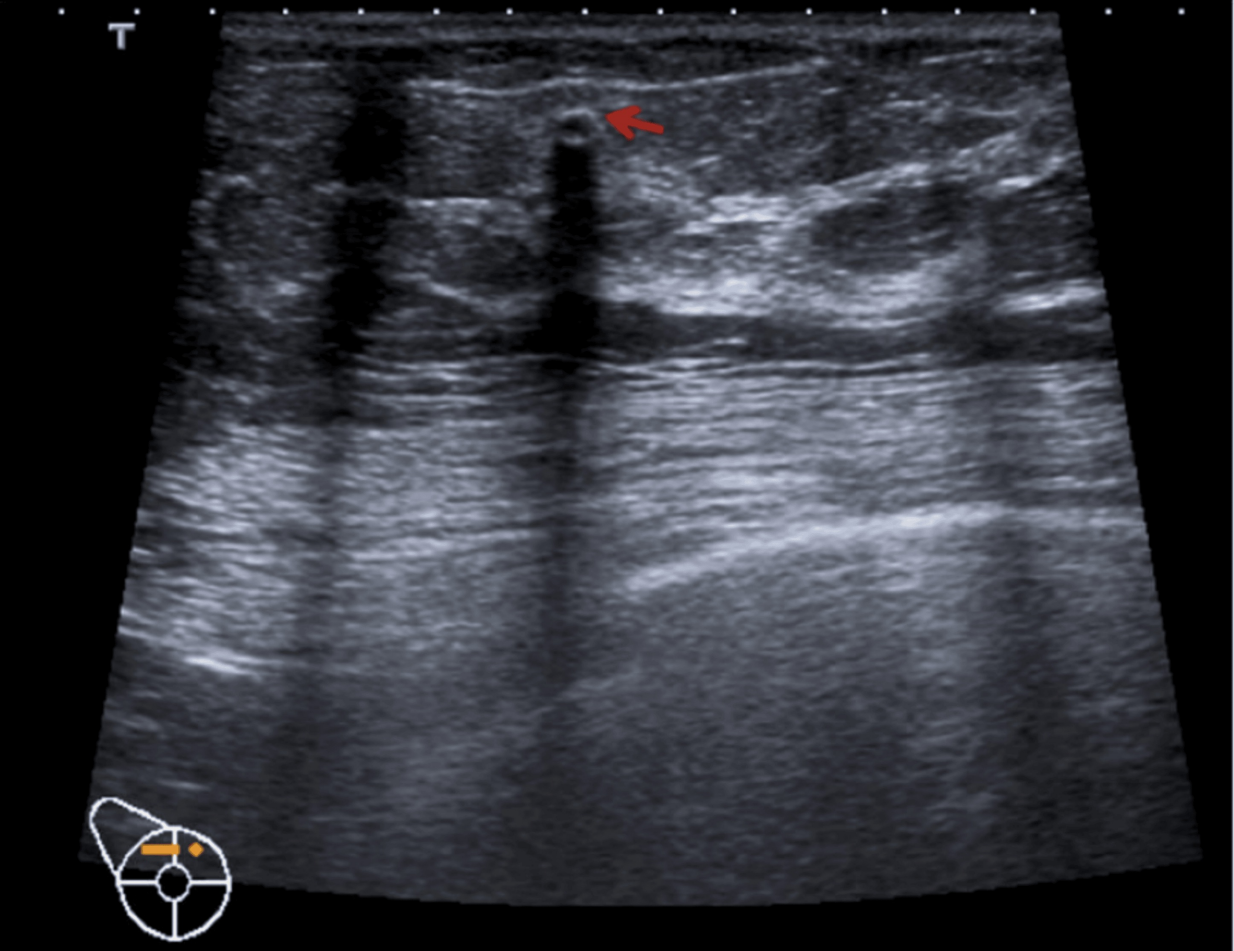
Breast ultrasound indicating fibrotic changes in the glandular tissue and a coarse calcification (red arrow) Image credit: Magalhaes et al. [43] (licensed under Creative Commons Attribution-NonCommercial-ShareAlike 4.0 International License; http://creativecommons.org/licenses/by-nc-sa/4.0/)

Figures 5, 6 show the LM images that visually depict common findings of calcification and fibrotic changes characteristic of the condition [43].

Discussion

LM is a subset of lupus panniculitis that impacts the subcutaneous layer of the breast as well as the mammary glands. LM is a rare condition that usually occurs in those with a previously established diagnosis of SLE or DLE; however, it rarely can also be the initial presentation [6].With its heterogeneous presentation, clinicians are unaware of this subsequent condition that can arise with a lupus diagnosis, as more prominent conditions often overshadow it.

In the diagnosis of LM, all cases received a combination of ultrasound, mammography, MRI, and rarely CT. Due to LM presenting physically similar to breast malignancy, it is crucial to make that differentiation. Non-invasive ultrasounds were encountered to be the most commonly utilized initial imaging. Ultrasound findings included hyperechoic, hypoechoic and isoechoic lesions with poorly defined borders, the masses are described as irregular, lobulated, or oval with acoustic shadowing, coarse strong echoes, and associated features of edema, increased vascularization, and skin thickening. Mammograms demonstrated ill-defined masses with high density, focal asymmetries, diffuse, coarse, or micro calcifications, and associated features of axillary lymphadenopathy on the side of insult and skin thickening. MRI findings included a type 1 kinetic curve correlating to benign findings, fat necrosis, rim-enhancing lesions, and diffuse and heterogeneous enhancement. The diagnosis of LM can become complicated as rim-enhancing lesions and heterogenous enhancement are both characteristics of malignancy. As a result, a previous diagnosis of lupus can help guide the diagnosis to LM [44,45]. Suspicious findings were deemed BIRADS 4 and biopsies were done following imaging, with more common pathological findings of lymphocytic, histiocytic, mononuclear cell infiltration, sclerosis, fibrosis, vasculitis, accumulation of mucin, and deposition of immune complexes. Lymphocytic infiltration has been deemed to be a key feature of LM; however, it is also commonly found within diabetic mastopathy. A key differentiating feature is that in LM, it is present in the lobules and is more profuse [5,7,35].

There are currently conflicting verdicts on the use of biopsy. Biopsies are not recommended unless imaging is inconclusive, as trauma to the site has been correlated to flare-ups; however, in almost all of the patients, biopsies were utilized following imaging [35,46]. This raises the question of the lack of consensus regarding diagnostic criteria for LM, resulting in the continuous use of biopsy to confirm the findings. However, some clinicians believe that the diagnosis of this condition should be conservative through clinical features, and imaging is sufficient to develop the diagnosis [16,47]. Surgical removal of the mass is further deemed unnecessary and was not completed in any of the studies analyzed. In the sparse cases in which gross examination was conducted, the masses were described as well-circumscribed, lobulated, irregular cavities with greasy liquid-filled, pink-tan nodular areas, yellow-tan adipose tissue with fibrous tissue, and white-tan induration [4,5].

Interestingly, six patients were not diagnosed with any form of lupus at the time of presentation. The reason for this matter can be due to LM not being a diagnostic criterion identified by the American College of Rheumatology. In order to be diagnosed with SLE based on the EULAR/ACR 2019 criteria, a score of above 10 is required, and an isolated incident of LM does not meet any criteria [48]. Furthermore, these patients may be in the preclinical stage of lupus with LM as an isolated symptom without yet meeting the rest of the criteria.

**Figure 7:**
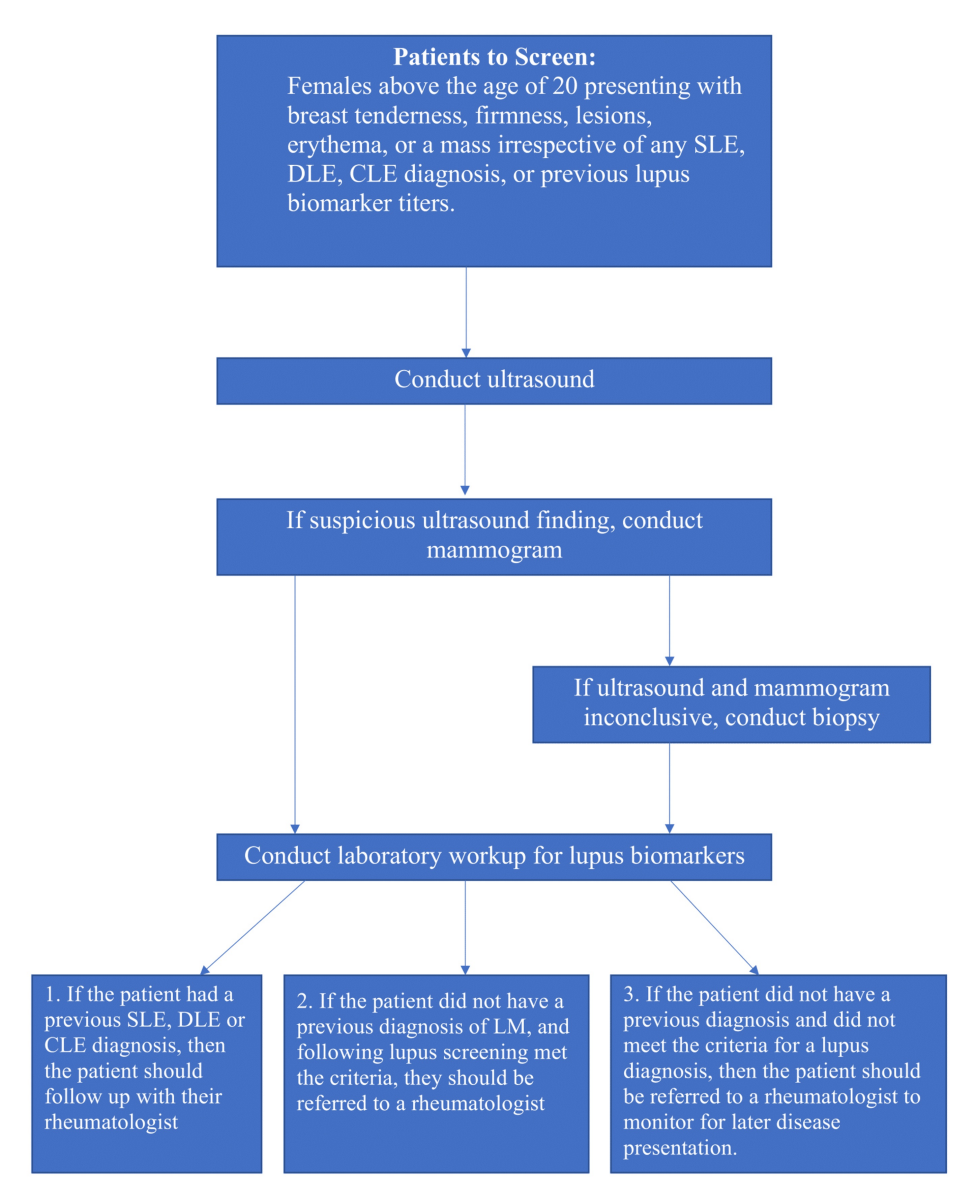
Suggested diagnostic management for LM SLE: Systemic lupus erythematosus; CLE: cutaneous lupus erythematosus; DLE: discoid lupus erythematosus; LM: lupus mastitis

Currently, no leading organization or committee has established published diagnostic criteria specifically for LM. Due to its heterogeneous presence and lack of guidance for clinicians, the diagnosis of LM is complex. LM is often misdiagnosed if it presents as firm, hard, palpable masses, or the calcifications seen within imaging can initially raise alarms for malignancy. Establishing precise diagnostic criteria is crucial to avoid unnecessary surgery and psychological worry among patients [7]. Through analyzing the case studies, we propose the first diagnostic and management guidelines and the following steps for the diagnosis of LM (Figure 7).

We also propose adapting current clinical guidelines to include the fact that a diagnosis of LM does satisfy the criteria of an SLE diagnosis with or without meeting the additional requirements required by established clinical guidelines. Similarly, after a renal biopsy indicates lupus nephritis, those patients are then subsequently diagnosed with lupus even if they do not meet the remaining (i.e. SLICC, ACR, EULAR) criteria. We propose a similar action be taken with LM. Thirty-six percent of patients were diagnosed with LM without having a previous diagnosis of lupus, and only four out of the 12 patients met diagnostic criteria for lupus, while the other eight did not but still had LM. The question is raised of how a patient could be diagnosed with LM without meeting the established criteria as a result, we believe they should be subsequently diagnosed with lupus and/or monitored to assess for subsequent development of SLE. Furthermore, we recommend patients who have not met established criteria for diagnosis to still be connected with a rheumatologist and follow up yearly, as LM may be the initial presentation of the condition that may present additional symptoms in the years to come. None of the case studies did follow-ups on the patient to monitor this phenomenon to see if they met lupus criteria later on as a result long-term and prospective cohort studies are recommended. 

The link between LM and traditional biomarkers of ANA, anti-dsDNA, anti-Sm, anti-histone, and complement factors of C3 and C4 is unclear in the literature. Only one external study analyzed these factors within “non-lactational mastitis,” but the patients did not meet the criteria for lupus. Within the case studies analyzed, a variety of biomarkers were analyzed, those found to be elevated amongst the patients included ANA, lupus anticoagulant, dsDNA antibodies, anti-SSA antibodies, anticardiolipin antibodies, and elevated IgA and IgG levels [6,9,13,15,22,25,26,40,41,49]. Additional biomarkers of interferon-alpha, IP-10, SIGLEC-1, CCL8, CXCL13, and IL-1 RA have been identified as biomarkers to indicate inflammatory states within SLE, but none have been investigated thus far in LM. Further research is required within the LM population to develop a clear understanding of the influence of lupus biomarkers; as patients are being diagnosed with LM without having elevated biomarkers, we recommend LM to be enough of an independent factor to diagnose a patient with lupus.

LM is a chronic disease that can result in remissions years later, as seen in almost 30% of the patient cases analyzed or if misdiagnosed, lead to prolonged care or disfigurement of the breast [3,5,6,21,22,27,34]. Lupus panniculitis which is the inflammation of the subcutaneous layer of the skin only requires corticosteroids either topically or as an injection; however, LM generally requires an anti-malarial medication of hydroxychloroquine that may be given individually or combined with steroids [5,6,11,15,21-27,32,33]. Hydroxychloroquine was first identified to be effective in CLE and has since expanded in its use for SLE [50].

Leading rheumatology organizations have yet to establish the causes and diagnostic criteria for LM. Given its rarity and similarity to malignancy in imaging, official guidelines are needed to assist clinicians in laboratory and imaging assessments

The limitations of this article include the scarce research conducted on patients with LM. As LM is a rare condition, the current evidence only consists of case studies, and there is a lack of primary studies conducted on these patients. Further research must be conducted to understand the underlying cause of LM, as that can guide diagnostic imaging and workup criteria. Furthermore, in patients who developed LM without meeting diagnostic criteria at the moment, research should be conducted to understand what patient characteristics may potentially lead to LM being the initial presentation of lupus in these patients. Finally, little demographic data was provided regarding the patients which should be further evaluated to better understand if a specific group of patients are at higher risk than others as trends are commonly seen within autoimmune conditions.

## Conclusions

LM is a complex condition that has been heavily under-researched in today’s literature. It has a heterogeneous presentation, occurs in various age ranges, and has a range of disease durations, and some patients who have not met the diagnostic criteria for SLE or DLE. Understanding this condition further is crucial to monitoring its development ahead of time and tailoring treatment options. A goal of this study was to provide a workflow for the diagnosis of LM and we strive that our recommended clinical guidelines will help provide clarity to clinicians on the imaging procedures to take when approached with the uncommon diagnosis of LM.

## References

[1] Kaposi M (1883). Pathologie und Therapie der Hautkrankheiten.

[2] Park HS, Choi JW, Kim BK, Cho KH (2010). Lupus erythematosus panniculitis: clinicopathological, immunophenotypic, and molecular studies. Am J Dermatopathol.

[3] Summers TA Jr, Lehman MB, Barner R, Royer MC (2009). Lupus mastitis: a clinicopathologic review and addition of a case. Adv Anat Pathol.

[4] De Bandt M, Meyer O, Grossin M, Kahn MF (1993). Lupus mastitis heralding systemic lupus erythematosus with antiphospholipid syndrome. J Rheumatol.

[5] Kinonen C, Gattuso P, Reddy VB (2010). Lupus mastitis: an uncommon complication of systemic or discoid lupus. Am J Surg Pathol.

[6] Voizard B, Lalonde L, Sanchez LM (2017). Lupus mastitis as a first manifestation of systemic disease: about two cases with a review of the literature. Eur J Radiol.

[7] Rosa M, Mohammadi A (2013). Lupus mastitis: a review. Ann Diagn Pathol.

[8] Fernandez-Flores A, Crespo LG, Alonso S, Montero MG (2006). Lupus mastitis in the male breast mimicking inflammatory carcinoma. Breast J.

[9] Crevits J, Van Steen A, Van Ongeval C, Marchal G (2009). Unilateral calcifying lupus mastitis in a male breast. Breast J.

[10] Martella S, Matthes AG, Bassi F, Fasani R, De Lorenzi F, Gatti G, Luini A (2008). Lupus mastitis in male mimicking a breast lump. Int J Surg.

[11] Thapa A, Parakh A, Arora J, Goel RK (2016). Lupus mastitis of the male breast. BJR Case Rep.

[12] Winkelmann RK (1983). Panniculitis in connective tissue disease. Arch Dermatol.

[13] Chen X, Hoda SA, Delellis RA, Seshan SV (2005). Lupus mastitis. Breast J.

[14] Castro GR, Appenzeller S, Soledade C, Bértolo MB, Costallat LT (2004). Mastitis refractory to cyclophosphamide in systemic lupus erythematosus. Clin Exp Rheumatol.

[15] Vilas-Sueiro A, González-Vilas D, Aguilera C, Monteagudo B, De Las Heras C (2017). Hardness and painful lesion of the breast. Acta Dermatovenerol Croat.

[16] Georgian-Smith D, Lawton TJ, Moe RE, Couser WG (2002). Lupus mastitis: radiologic and pathologic features. AJR Am J Roentgenol.

[17] Cerveira I, Costa Matos L, Garrido A (2006). Lupus mastitis. Breast.

[18] Vaglio A, Grayson PC, Fenaroli P, Gianfreda D, Boccaletti V, Ghiggeri GM, Moroni G (2018). Drug-induced lupus: traditional and new concepts. Autoimmun Rev.

[19] Xiao X, Chang C (2014). Diagnosis and classification of drug-induced autoimmunity (DIA). J Autoimmun.

[20] Page MJ, McKenzie JE, Bossuyt PM (2021). The PRISMA 2020 statement: an updated guideline for reporting systematic reviews. Syst Rev.

[21] Guo CY, Chen L, Sun L (2023). Rare and first manifestation of lupus panniculitis as lupus mastitis: a case report and literature review. Curr Med Imaging.

[22] Taslicay CA, Dervisoglu E, Yaprak Bayrak B, Mese I, Arslan AS (2023). A mimicker of inflammatory breast carcinoma: lupus mastitis and evolving imaging features. J Clin Ultrasound.

[23] Wang Y, Feng Z, Ma B, Li S, Guo W, Song J, Yan Z (2024). Diagnosis of lupus mastitis via multimodal ultrasound: case description. Quant Imaging Med Surg.

[24] Pimentel A, Moreira A, Silva S, Lages R, Noronha J (2021). Lupus mastitis: a rare breast cancer differential diagnosis. Clin Case Rep.

[25] Oktay A, Esmat HA, Aslan Ö, Mirzafarli I (2022). Lupus mastitis in a young female mimicking a breast carcinoma: a rare entity through a case report and review of the literature. Eur J Breast Health.

[26] Jiménez-Antón A, Jiménez-Gallo D, Millán-Cayetano JF, Navarro-Navarro I, Linares-Barrios M (2023). Unilateral lupus mastitis. Lupus.

[27] Mazeda C, Aguiar R, Barcelos A (2023). Lupus mastitis - the copycat. Scand J Rheumatol.

[28] Sharma A, Blank A, Komforti MK (2021). Rare initial manifestation of lupus as lobular panniculitis of the breast—a case report and review of the literature. Am J Dermatopathol.

[29] Tanaka Y, Manabe H, Shinzaki W, Hashimoto Y, Komoike Y (2020). A case of lupus mastitis in a patient with systemic lupus erythematosus. Breast J.

[30] Kim S, Klein N (2024). Lupus mastitis: the rare mimic of breast malignancy. J Breast Imaging.

[31] Lucivero G, Romano C, Ferraraccio F (2011). Lupus mastitis in systemic lupus erythematosus: a rare condition requiring a minimally invasive diagnostic approach. Int J Immunopathol Pharmacol.

[32] Wang YC, Chou CP, Levenson RB, Hsieh PP, Huang JS, Pan HB (2010). Imaging features of bilateral lupus mastitis. Breast J.

[33] Mosier AD, Boldt B, Keylock J, Smith DV, Graham J (2013). Serial MR findings and comprehensive review of bilateral lupus mastitis with an additional case report. J Radiol Case Rep.

[34] Warne RR, Taylor D, Segal A, Irish A (2011). Lupus mastitis: a mimicker of breast carcinoma. BMJ Case Rep.

[35] Wani AM, Mohd Hussain W, Fatani MI, Shakour BA (2009). Lupus mastitis - peculiar radiological and pathological features. Indian J Radiol Imaging.

[36] Arsenovic N, Terzic M (2008). Lupus mastitis mimicking a breast tumor. J Obstet Gynaecol Res.

[37] Sanders LM, Lacz NL, Blackwood MM, Ongcapin E, Santos-Zabala ML (2012). Lupus mastitis without systemic disease: unusual imaging findings including MRI. Breast J.

[38] Fernández-Torres R, Sacristán F, Del Pozo J, Martínez W, Albaina L, Mazaira M, Fonseca E (2009). Lupus mastitis, a mimicker of erysipelatoides breast carcinoma. J Am Acad Dermatol.

[39] Bachmeyer C, Goubin I, Berseneff H, Blum L (2006). Coarse calcifications by mammography in lupus mastitis. Arch Dermatol.

[40] Nigar E, Contractor K, Singhal H, Matin RN (2007). Lupus mastitis - a cause of recurrent breast lumps. Histopathology.

[41] Carducci M, Mussi A, Lisi S, Muscardin L, Solivetti FM (2005). Lupus mastitis: a 2-year history of a single localization of lupus erythematosus mimicking breast carcinoma. J Eur Acad Dermatol Venereol.

[42] Sabaté JM, Gómez A, Torrubia S, Salinas T, Clotet M, Lerma E (2006). Lupus panniculitis involving the breast. Eur Radiol.

[43] Magalhaes SP, Macedo C, Alves N, Certo M, Reis F (2016). Lupus Mastitis - rare but typical imaging findings. Eur Soc Rad.

[44] Schnall MD, Blume J, Bluemke DA (2006). Diagnostic architectural and dynamic features at breast MR imaging: multicenter study. Radiology.

[45] Macura KJ, Ouwerkerk R, Jacobs MA, Bluemke DA (2006). Patterns of enhancement on breast MR images: interpretation and imaging pitfalls. Radiographics.

[46] Ujiie H, Shimizu T, Ito M, Arita K, Shimizu H (2006). Lupus erythematosus profundus successfully treated with dapsone: review of the literature. Arch Dermatol.

[47] Reginatto A, Pedrini J, Schorr M (2012). Report of lupus mastitis. SIS.

[48] Aringer M, Costenbader K, Daikh D (2019). 2019 European League Against Rheumatism/American College of Rheumatology Classification Criteria for Systemic Lupus Erythematosus. Arthritis Rheumatol.

[49] Chatterjee S, Caporale A, Tao JQ (2021). Acute e-cig inhalation impacts vascular health: a study in smoking naïve subjects. Am J Physiol Heart Circ Physiol.

[50] Shipman WD, Vernice NA, Demetres M, Jorizzo JL (2020). An update on the use of hydroxychloroquine in cutaneous lupus erythematosus: a systematic review. J Am Acad Dermatol.

